# Treatment of loiasis: a review of clinical management recommendations

**DOI:** 10.1186/s40249-025-01300-0

**Published:** 2025-05-07

**Authors:** Dorothea Ekoka Mbassi, Rike Dreeßen, Rella Zoleko Manego, Saskia Dede Davi, Tamara Nordmann, Johannes Mischlinger, Michael Ramharter

**Affiliations:** 1 Center for Tropical Medicine, Bernhard Nocht Institute for Tropical Medicine and I. Department of Medicine, University Medical Center Hamburg-Eppendorf, Hamburg, Germany; 2 German Center for Infection Research (DZIF), Partner Site Hamburg-Lübeck-Borstel-Riems, Hamburg, Germany; 3 Centre de Recherches Médicales de Lambaréné (CERMEL), Lambaréné, Gabon

**Keywords:** Loiasis, *Loa loa*, Treatment, Guideline

## Abstract

**Background:**

Loiasis affects more than 20 million residents of endemic regions in Central and West Africa causing chronic and often lifelong disease. Antifilarial treatment options for loiasis include diethylcarbamazine, ivermectin, and albendazole. Safe and effective management requires classifying patients into occult, microfilaremia, and hypermicrofilaremia categories. Treatment is complicated by the risk of severe adverse events, particularly encephalitis. Clear guidance on the appropriate use of antifilarial therapy is therefore of utmost importance. The aim of this review is to evaluate current treatment recommendations and assess their quality and consistency.

**Methods:**

A scoping review was conducted to evaluate treatment recommendations for loiasis. The literature search encompassed multiple databases, including PubMed and specialized medical repositories, without restrictions on publication date or language. The approach included a systematic search with specific loiasis-related keywords and an unstructured search of guidelines from health ministries in endemic countries, along with grey literature and professional recommendations. Renowned tropical medicine textbooks were also consulted. Data were extracted with a detailed table collaboratively developed and reviewed by multiple researchers to ensure consistency and accuracy.

**Results:**

The review identified 33 sources, consisting of nine guidelines, ten reviews, and 14 textbook excerpts. Publications reviewed spanned from 2001 to 2024 with no major innovations in treatment noted. Evidence quality was often low, with only two guidelines detailing their development process. Variability was particularly noted in dosage protocols for diethylcarbamazine, typically dosed incrementally. Ivermectin and albendazole were mostly noted as alternatives based on microfilarial levels. The common microfilarial threshold was 8000 microfilariae per millilitre, dictating treatment strategy adjustments. Adjunctive treatments, such as corticosteroids and antihistamines, were inconsistently proposed to mitigate side effects.

**Conclusions:**

Inconsistencies between some recommendations were observed. There is an urgent need for internationally harmonized, evidence-based guidelines to address these inconsistencies, improve patient outcomes and minimize treatment-associated severe adverse events and fatalities.

**Graphical Abstract:**

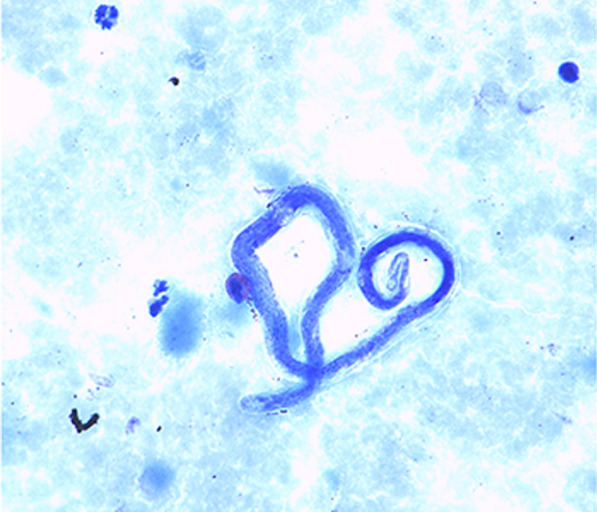

## Background

*Loa loa* affects more than 20 million people in the endemic regions of sub-Saharan Africa. It is transmitted by blood-sucking *Chrysops* flies and leads to chronic infections characterized by Calabar swelling, migration of the adult worm through the conjunctiva of the eye, pruritus, and occasionally complications in multiple organ systems, including cardiac, respiratory, neurological, ophthalmic, renal, gastrointestinal, and dermatological manifestations. Diagnostic challenges persist, with diagnosis in endemic settings often relying only on thick smear microscopy. However, only about one third of infected individuals show detectable microfilaremia. The three drugs mainly recommended for loiasis treatment are diethylcarbamazine, ivermectin, and albendazole [1].

Classifying patients into the categories of occult, microfilaremic, and hypermicrofilaremic infection is essential for appropriate loiasis treatment decisions [2] to avoid the risk for treatment-associated severe neurological adverse events (AEs) or fatalities which is linked to microfilarial load. Occult infection is marked by indirect signs and symptoms of loiasis, such as eye worm migration and Calabar swelling due to adult worm movements, occurring without detectable microfilaremia [1]. Hypermicrofilaremia is particularly concerning due to its predisposition to severe side effects from rapidly acting drugs such as diethylcarbamazine and ivermectin, with encephalitis being the most severe AE. [3] This underscores the necessity for unambiguous therapeutic protocols that prevent incorrect allocation of therapeutic regimens.

Clear and harmonized guidelines are needed to minimize severe adverse events and deaths associated with therapy, especially given the complexity of treatment algorithms. These complexities include the influence of microfilarial levels, the contraindication of diethylcarbamazine in individuals coinfected with onchocerciasis [4], and the potential complications, such as encephalitis, in hypermicrofilaremic patients. Establishing such guidelines would help minimize therapy-associated severe AEs and deaths and to reduce the considerable burden caused by loiasis [5].

While more clinical trials are needed to establish evidence-based treatment regimens, harmonized guidelines are already an essential tool for clinicians to effectively treat their patients. This approach could reduce the use of drug regimens that lack a strong evidence base. Therefore, not only are more clinical trials needed, but their results should also be integrated into clinical practice through updated guidelines.

This review aims to compile national and international guidelines, as well as recommendations by scientific institutions and associations, to assess the available loiasis treatment guidelines for their recommendations, consistency and quality of evidence. This review therefore focusses on individual treatment protocols and not on potential recommendations for mass drug administration programs to reduce transmission on a population level.

## Methods

### Search strategy, inclusion and exclusion criteria

First, a systematic literature search was performed, followed by an unstructured literature search. Only guidelines, manuals, reviews and textbook excerpts were eligible for this review. The systematic literature search was performed on PubMed via the search term ‘("loa loa" OR loiasis OR loaose OR Loíase) AND (guideline OR recommendation)’ and not restricted by publication date or language. Then, a search with the search term ‘Loa OR "loa loa" OR loiasis OR loaose OR Loíase’ was performed on Trip Medical Database, Guideline Central, Guidelines International Network, JAMA Evidence, and the Harvard Medical School Library of Evidence.

In the following, unstructured literature search, we used different approaches. For endemic countries, a search for loiasis treatment guidelines was conducted on the websites of health ministries. Additional sources that discuss treatment recommendations were identified via Google search, personal literature collections and professional connections. This was supposed to yield scientific publications not found via the systematic search, as well as grey literature. Lastly, renowned textbooks in the field of Tropical Medicine from the libraries of the Bernhard Nocht Institute for Tropical Medicine and the Central Medical Library of Hamburg were screened for specific treatment recommendations. During this search stage, eligible languages were English, German and French due to the authors’ language skills.

### Quality assessment of included literature

The quality of the included literature was assessed by reviewing the presence of evidence grading systems and detailed methodologies within the sources.

### Evidence extraction and analysis

A table was collaboratively developed by two reviewers to capture information during the literature search. Initially, one reviewer conducted the literature search, and it was later updated and refined by another to enhance accuracy. The process was supervised by a third author, with guidance provided on where and how to search for additional sources. This iterative approach ensured the data collection template effectively captured all relevant variables.

### Gap analysis

The gap analysis involved identifying inconsistencies and omissions across different guidelines, focusing on the lack of standard methodologies and consistent microfilarial thresholds, to pinpoint areas requiring further research and development.

## Results

**Fig. 1:**
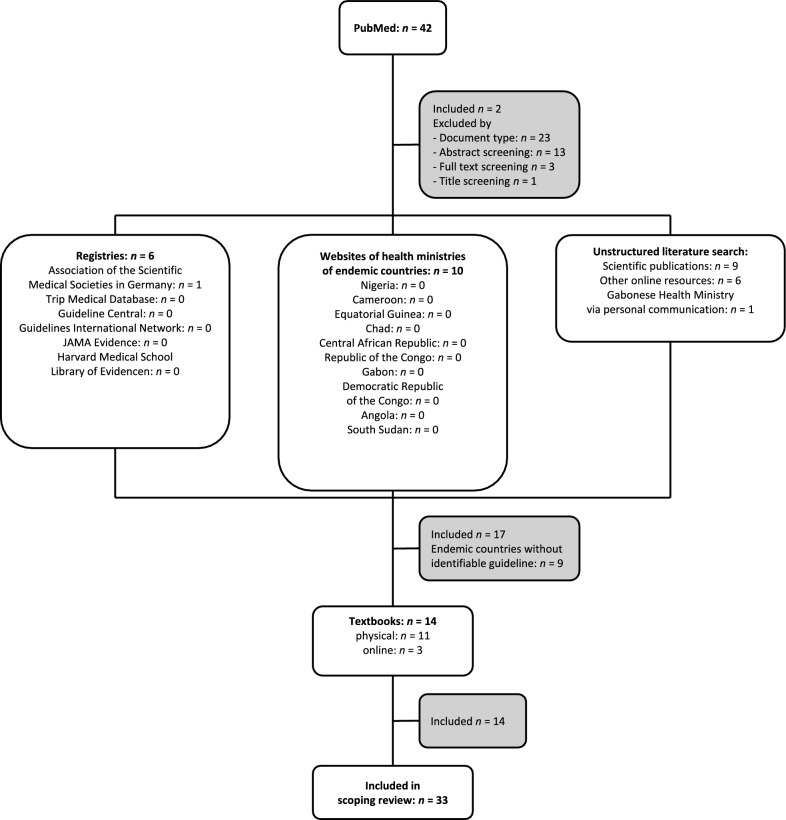
Flow of literature search

The search performed yielded 33 sources: nine guidelines, ten reviews, and 14 textbook excerpts (Fig. 1, Table 1). Among the searched guideline repositories, only the Association of the Scientific Medical Societies in Germany offers specific guidelines on loiasis therapy. Country-level guidelines were identified from Gabon, Germany and the United Kingdom [6–8]. Several guidelines from the United States of America are aimed at practitioners worldwide [9–12].

**Table 1:** Recommendations for the treatment of loiasis

Population	Occult loiasis	Microfilaremic infection	Hypermicrofilaremic infection	Other	Reference, Year
Guidelines					
Endemic		< 2000 mf/ml, adults*: Diethylcarbamazine 200 mg twice daily for 28 days (gradual dose increase from 6 mg twice daily), second course four weeks later if needed < 2000 mf/ml, children*: Diethylcarbamazine 1.5 mg/kg twice daily for 28 days (from gradual dose increase)	< 8000 mf/ml: Ivermectin 150 μg/kg single-dose, monthly repeat if needed, then diethylcarbamazine < 30,000 mf/ml, diethylcarbamazine failure or contraindication: Ivermectin 150 μg/kg single-dose, monthly or quarterly repeat if needed > 30,000 mf/ml: Possibly albendazole 200 mg twice daily for 21 days, then ivermectin 150 μg/kg once daily for five days (inpatient), then diethylcarbamazine	Eye migration: Surgical removal not recommended Adjunct therapy: Paracetamol for first seven days for treatment of hypermicrofilaremic infection	Médecins Sans Frontières [14], 2024
Germany: travellers and migrants	Diethylcarbamazine 3 mg/kg three times daily for 21 days, repeat if needed Or albendazole 400 mg twice daily for 28 days Or albendazole, then ivermectin 150–200 µg/kg single-dose	< 2000 mf/ml: Diethylcarbamazine (gradual dose increase from 50 mg/d) Or albendazole Or albendazole, then ivermectin < 8000 mf/ml: Albendazole, then diethylcarbamazine Or ivermectin, then diethylcarbamazine Or albendazole, then ivermectin, then diethylcarbamazine	< 30,000 mf/ml: Possibly apheresis, then ivermectin or albendazole or diethylcarbamazine Or albendazole 400 mg once or twice daily for 28 days, then ivermectin or diethylcarbamazine > 30,000 mf/ml: Apheresis, then ivermectin or albendazole or diethylcarbamazine Or albendazole (unclear dosage)	Prophylaxis: Diethylcarbamazine 300 mg weekly Adjunct therapy: Possibly antihistamines or corticosteroids	Association of the Scientific Medical Societies in Germany [7], 2024
Endemic, and travellers and migrants	Diethylcarbamazine 3 mg/kg three times daily for 21 days†,	< 2500 mf/ml, *O. volvulus* coinfection: Ivermectin inconclusive recommendation Diethylcarbamazine for 21 days (gradual dose increase from 50 to 1 mg/kg), possibly after pre-therapy, repeat if needed†	> 2500 mf/ml or diethylcarbamazine failure, and symptomatic: Albendazole 200 mg twice daily for 21 days, repeat if needed, then diethylcarbamazine > 2500 mf/ml and symptomatic: Apheresis, then diethylcarbamazine	Prophylaxis: Diethylcarbamazine 300 mg weekly Eye migration: Surgical removal only for diagnostic purposes or intraocular worm Adjunct therapy: Possibly concomitant corticosteroids or antihistamines	UpToDate [9], 2022
UK: travellers and migrants	Diethylcarbamazine‡ 200 mg three times daily for 21 days (gradual dose increase from 50 mg daily)	< 1000 mf/ml: Diethylcarbamazine‡	> 1000 mf/ml: Albendazole 200 mg twice daily for 21 days, then diethylcarbamazine	Adjunct therapy: prednisolone 30 mg once daily for 7 days, starting the day before diethylcarbamazine	UK guideline [8], 2024
USA: endemic, and travellers and migrants		Laboratory-confirmed loiasis < 8000 mf/ml: Diethylcarbamazine, 2.7 to 3.3 mg/kg three times daily for 21 days, repeat if needed ‡	> 8000 mf/ml or diethylcarbamazine failure: Albendazole 200 mg twice daily for 21 days, possibly also apheresis, then diethylcarbamazine > 8000 mf/ml: Apheresis, possibly also albendazole, then diethylcarbamazine	Prophylaxis: Diethylcarbamazine 300 mg once weekly	Merck Sharp & Dohme manual [11], 2022
Gabon: endemic		< 8000 mf/ml: Ivermectin 200 μg/kg daily for 10 days	> 8000 mf/ml: Albendazole 800 mg daily for 10 days	Mass treatment: Unknown population microfilaremia: Albendazole 400 mg daily for 21 days Population microfilaremia < 8000 mf/ml: Ivermectin 200 μg/kg single dose Population microfilaremia > 8000 mf/ml: Albendazole 400 mg daily for 21 days Adjunct therapy: Concomitant corticosteroids (e.g., prednisone 0.5 mg/kg for 5 days)	Gabonese health ministry [6], year unknown
USA: travellers and migrants		< 8000 mf/ml and symptoms: Diethylcarbamazine 2.7–3.3 mg/kg three times daily for 21 days, one or two courses‡ < 8000 mf/ml and diethylcarbamazine failure (twice) or contraindication: Albendazole 200 mg twice daily for 21 days§	> 8000 mf/ml and symptoms: Apheresis or albendazole, then diethylcarbamazine	Adjunct therapy: possibly concomitant corticosteroids or antihistamines	U.S. Centers for Disease Control and Prevention [12], 2020
USA: endemic, and travellers and migrants	Diethylcarbamazine 400 mg daily (or 8–10 mg/kg per day) for 21–28 days (gradual dose increase from 50 mg daily), divided into 2–3 doses, repeat if needed	< 2000 mf/ml: Diethylcarbamazine 400 mg daily (or 8–10 mg/kg per day) for 21–28 days (gradual dose increase from 3 or 6 mg daily), divided into 2–3 doses, repeat if needed Or albendazole 200 mg twice daily for 21 days or ivermectin < 8000 mf/ml: Ivermectin 150 µg/kg single dose, repetition every 1–3 months, if needed, then diethylcarbamazine	< 30,000 mf/ml: Ivermectin or albendazole, then diethylcarbamazine > 30,000 mf/ml: Albendazole or apheresis, then diethylcarbamazine	Prophylaxis: Diethylcarbamazine 300 mg once weekly or 200 mg twice daily for 3 days monthly Eye migration: Surgical removal Adjunct therapy: Possibly concomitant antihistamines or corticosteroids	Medscape [10], 2020
France: endemic, and travellers and migrants		< 30,000 mf/ml: Diethylcarbamazine for several weeks < 30,000 mf/ml: Ivermectin single dose, after albendazole pre-therapy	Albendazole, then diethylcarbamazine or ivermectin		French health ministry [33], 2018
Reviews					
Endemic, and travellers and migrants	Diethylcarbamazine 9 mg/kg in three divided doses daily for 21 days, repeat if necessary Or albendazole 400 mg twice daily for 28 days, possibly followed by ivermectin 150–200 µg/kg single dose	< 2000 mf/ml: diethylcarbamazine (gradual dose increase, starting dose 50 mg), repeat if necessary Or albendazole, possibly followed by ivermectin < 8000 mf/ml: Albendazole, possibly followed by diethylcarbamazine Or ivermectin followed by diethylcarbamazine Or albendazole, followed by ivermectin, possibly followed by diethylcarbamazine	< 30,000 mf/ml: Albendazole, possibly followed by ivermectin, possibly followed by diethylcarbamazine > 30,000 mf/ml: Apheresis, followed by chemotherapy Or albendazole, followed by chemotherapy	Adjunct treatments: possibly antihistamines and corticosteroids	[1], 2024
Italy: travellers and migrants		< 8000 mf/ml: Diethylcarbamazine 8–10mg/kg per day in divided doses for 21 days, possibly one repetition, then albendazole Or possibly imatinib‡ Co-infection with onchocerciasis: Ivermectin 150 µg/kg single dose	> 8000 mf/ml: Albendazole 200 mg twice daily for 21 days, then diethylcarbamazine Or apheresis Or possibly imatinib	Adjunct treatments: Prednisone (start with 60 mg/d) Mention of reslizumab	[21], 2022
Endemic, and travellers and migrants	No threshold: Albendazole 800 mg daily for 28 days, then ivermectin Or mebendazole 300–1500 mg daily for 21 days, then ivermectin or diethylcarbamazine				[31], 2021
Endemic, and travellers and migrants	Across populations: diethylcarbamazine (most frequently), mebendazole, And imatinib	< 8000 mf/ml: Ivermectin	> 8000 mf/ml: albendazole		[17], 2019
USA: travellers and migrants		< 8000 mf/ml: Diethylcarbamazine	> 8000 mf/ml: Adjunct/second-line cytapheresis, then diethylcarbamazine		[32], 2018
Endemic, and travellers and migrants		< 20,000–30,000 mf/ml: Diethylcarbamazine or ivermectin	> 20,000–30,000 mf/ml: albendazole, then ivermectin		[34], 2018
France/travellers and migrants	Diethylcarbamazine 400 mg/d divided in 2–3 daily doses for 21 to 28 days (progressive dose increase from 50 mg/d), repeat if needed, possibly followed by albendazole 200 mg twice daily for 21 days	< 2000 mf/ml: Diethylcarbamazine 400 mg/d divided in 2–3 daily doses for 21 to 28 days (progressive dose increase from 3–6 mg/d), repeat if needed, possibly followed by albendazole 200 mg twice daily for 21 days < 8000 mf/ml: Ivermectin 150 µg/kg single dose. Courses every 1–3 months if needed, followed by diethylcarbamazine	< 30,000 mf/ml: Ivermectin under hospitalization Or albendazole followed by ivermectin > 30,000 mf/ml: Albendazole, possibly apheresis	Adjunct therapy: Possibly concomitant corticosteroids or antihistamines	[29], 2012
Switzerland: endemic, and travellers and migrants		< 100 mf/ml: Diethylcarbamazine 9 mg/kg daily for 21 days (gradual dose increase from 1 mg/kg) > 100 mf/ml: Albendazole 100 mg twice daily, three times a week < 1000 mf/ml: Diethylcarbamazine 9 mg/kg for 21 days (gradual dose increase from 1 mg/kg), possibly after albendazole and/or ivermectin pre-therapy < 8000 mf/ml: Ivermectin 150–200 μg/kg single dose, possibly after albendazole pre-therapy	> 8000 mf/ml: Albendazole 200 mg twice daily for 21 days, as pre-therapy > 8000 mf/ml: Apheresis as pre-therapy	Adjunct therapy: possibly concomitant corticosteroid or antihistamines	[25], 2012
Endemic, and travellers and migrants		< 1000 mf/ml: Diethylcarbamazine 300–400 mg/d (gradual dose increase from 6.25 or 12.5mg/d) for 21 to 28 days < 8000 mf/ml: Ivermectin 150–200 µg/kg single dose, then diethylcarbamazine	> 8000 mf/ml: Albendazole 200 mg twice daily for 21 days, or apheresis, then diethylcarbamazine 30,000–50,000 mf/ml: Albendazole or apheresis, then diethylcarbamazine	Prophylaxis: Diethylcarbamazine 300 mg weekly or 5 mg/kg for three consecutive days monthly Adjunct therapy: Possibly concomitant corticosteroids or antihistamines	[18], 2006
Endemic		< 50,000 mf/ml: Ivermectin 200 µg/kg single dose, repeat every 6 months if needed		Diethylcarbamazine not further mentioned because of unavailability	[13], 2001
Textbooks					
Endemic		< 8000 mf/ml: Diethylcarbamazine 8 mg/kg three times daily for 21 days (gradual dose increase from 50 mg) < 20,000 mf/ml: Ivermectin 150–200 μg/kg single dose, possibly with diethylcarbamazine or albendazole	Albendazole 200–400 mg orally twice daily for 21 days		*Loa loa*: latest advances in loiasis research (Akue) [2], 2024
Endemic, and travellers and migrants		< 8000 mf/ml: Diethylcarbamazine 5–10 mg/kg daily in divided doses for 14 to 28 days, repeat if needed ‡ Or ivermectin 200 mg/kg	> 8000 mf/ml: Apheresis, then diethylcarbamazine	Prophylaxis: Diethylcarbamazine 300 mg weekly	Manson’s tropical diseases [30], 2023
Endemic, and travellers and migrants	Diethylcarbamazine 8–10 mg/kg orally for 21 days, repeat if needed Or ivermectin or albendazole	< 30,000 mf/ml: Diethylcarbamazine 8–10 mg/kg orally for 21 days, repeat if needed or ivermectin or albendazole	> 30,000 mf/ml: Apheresis and/or glucocorticoids (40–60 mg prednisone daily, possibly tapered rapidly), then diethylcarbamazine 8–10 mg/kg daily (gradual dose increase from 0.5 mg/kg daily) or albendazole	Prophylaxis: diethylcarbamazine 300 mg weekly	Harrison’s principles of internal medicine [22], 2022
Endemic, and travellers and migrants	Diethylcarbamazine 400 mg/d for 21–28 days (gradual dose increase from 50 mg/d)	< 2000 mf/ml: diethylcarbamazine (gradual dose increase from 3–6 mg/d) < 8000 mf/ml: Ivermectin 150 µg/kg single dose every 1–3 months, then diethylcarbamazine	< 10,000 mf/ml: See < 8000 mf/ml, inpatient care < 30,000 mf/ml: albendazole 200mg twice daily for 21 days, then ivermectin or diethylcarbamazine > 30,000 mf/ml: albendazole or apheresis, then ivermectin or diethylcarbamazine	Prophylaxis: diethylcarbamazine 200 mg twice daily for three days monthly or 300 mg weekly	ePILLY [24], 2022
Endemic, and travellers and migrants	Diethylcarbamazine 9 mg/kg for 14 to 28 days (gradual dose increase from 6 mg/kg)	< 1000 mf/ml: Diethylcarbamazine for 14 to 28 days (inpatient, gradual dose increase from 1 mg/kg) < 8000 mf/ml: Ivermectin 150–200 μg/kg single-dose	> 8000 mf/ml: Apheresis, then ivermectin or diethylcarbamazine‡ > 8000 mf/ml, *O. volvulus* coinfection: Albendazole 200 mg twice daily for 21 days or mebendazole, then ivermectin or diethylcarbamazine	Eye migration: Surgical removal Adjunct therapy: Possibly concomitant salicylates, antihistamines or steroids (if microfilaremia > 25 mf/ml)	Meyer Tropenmedizin [26], 2021
Endemic		< 2500 mf/ml: Diethylcarbamazine for 21 days (gradual dose increase), repeat if needed Or albendazole	> 2500 mf/ml: Apheresis or albendazole several weeks	Prophylaxis: Diethylcarbamazine 300 mg weekly Eye migration: surgical extraction possible Adjunct therapy: Possibly concomitant antihistamines or corticosteroids	Parasitic diseases (parasites without borders) [35], 2019
Switzerland: endemic, and travellers and migrants		< 1000 mf/ml, adults: Diethylcarbamazine 150 mg three times daily or 9 mg/kg daily for 21 days (gradual dose increase from 25 mg single dose) < 1000 mf/ml, children: Diethylcarbamazine 3 mg/kg three times daily for 21 days (gradual dose increase from 0.5 mg/kg single dose) > 1000 mf/ml: Albendazole 200 mg twice daily for 21 days, then diethylcarbamazine	> 8000 mf/ml: Possibly apheresis / plasmapheresis, then albendazole or diethylcarbamazine	Diethylcarbamazine failure: Albendazole 200 to 400 mg twice daily for 21 to 28 days Adjunct therapy: Concomitant corticosteroids or antihistamines	Antiparasitic treatment recommendations [15], 2018
Germany: travellers and migrants	Diethylcarbamazine and ivermectin	Diethylcarbamazine and ivermectin	Albendazole, then diethylcarbamazine and ivermectin, inpatient, possibly concomitant antihistamines and corticosteroids	Eye migration: possibly surgical extraction	Medizinische Mikrobiologie und Infektiologie (Suerbaum) [36], 2016
USA: endemic, and travellers and migrants		Diethylcarbamazine 6–9 mg/kg daily for 21 days (gradual dose increase from 1 mg/kg daily)	Diethylcarbamazine 6–9 mg/kg daily for 21 days (gradual dose increase from 1 mg/kg daily), concomitant corticosteroids for 2–3 days	If *O. volvulus* coinfection: Ivermectin 150 µg/kg, then diethylcarbamazine Prophylaxis: Diethylcarbamazine 300 mg once weekly	Oxford handbook of tropical medicine [27], 2014
Endemic, and travellers and migrants		Irrespective of microfilaremia: diethylcarbamazine 6 mg daily for 14 to 21 days Or ivermectin 150 µg/kg single-dose			Antibiotika-Therapie (Stille) [16], 2013
Endemic, and travellers and migrants		< 1000 mf/ml: diethylcarbamazine 300–400 mg/d for 21 to 28 days (gradual dose increase from 6.25–12.5 mg), repetition every two or three weeks if needed < 8000 mf/ml: Ivermectin 150–200 µg/kg single dose, then diethylcarbamazine	> 8000 mf/ml: Albendazole 200 mg twice daily for 21 days, then ivermectin, then diethylcarbamazine		Principles of medicine in Africa [20], 2013
France: endemic, and travellers and migrants	Diethylcarbamazine 400 mg/d for 21 days (gradual dose increase from 50 mg twice daily), repeat 10 d/month for 3–6 months, if needed	Diethylcarbamazine 400 mg/d for 21 days (gradual dose increase from 6.25 mg twice daily), repeat if needed	< 30,000 mf/ml: ivermectin or albendazole, then diethylcarbamazine	Prophylaxis: Diethylcarbamazine 50 mg twice weekly or 100 mg weekly Adjunct therapy: Concomitant antihistamines and corticosteroids (15–20 mg prednisone/d),	Médecine tropicale [19], 2012
Germany: endemic, and travellers and migrants		< 1000 mf/ml: Diethylcarbamazine 9 mg/kg for 21 days (gradual dose increase from 1 mg/kg) < 8000 mf/ml: ivermectin 150–200 µg/kg single dose	< 8000 mf/ml: albendazole 200 mg twice daily for 21 days or apheresis	Adjunct therapy: Possibly concomitant antihistamines or corticosteroids	Tropenmedizin in Klinik und Praxis (Löscher and Burchard) [28], 2010
USA: endemic, and travellers and migrants		Diethylcarbamazine 6 mg/kg daily For 12 days Or ivermectin	Albendazole or ivermectin	Prophylaxis: Diethylcarbamazine 300 mg once weekly Eye migration: Surgical removal, then systemic therapy	Stanford [23], 2009

^*^Not for pregnant women, infants, and patients in poor general condition, *O. volvulus* co-infection

†Not for asymptomatic patients, pregnant women, *O. volvulus* coinfection

‡Not in *O. volvulus* coinfection

§Not for infants until 1 year of age, pregnant women in first trimester

*(Onchocerca volvulus* = *O. volvulus)*

The recommendations date from 2001 to 2024, with no major changes or new treatment options observed. All publications that did not specify whether they applied to endemic populations or to travellers and migrants were considered applying to both. Of the 33 sources examined, five offered recommendations specifically for treatment in endemic areas, while seven focused solely on returning travellers and migrants. 21 sources provided guidance applicable to both populations. Among the medical guidelines, two addressed only endemic populations, and three focused exclusively on returning travellers and migrants, whereas four covered both groups. In terms of reviews, one was implicitly dedicated to treatment in endemic regions [13], three concentrated on the treatment of returning travellers and migrants, and six addressed both demographics. When considering textbook excerpts, two were specific to endemic regions, one was dedicated to travellers and migrants, and eleven covered both populations. While all selected sources provided recommendations for treating *L. loa* infection, eleven also offered guidelines on prophylactic therapy, and six made recommendations for managing cases of eye migration. Additionally, one source suggested a treatment regimen suitable for mass drug administration [6]. Four sources considered treatment restrictions for pregnant women or children [9, 12, 14, 15].

### Methodology and quality of publications

The evidence-base, quality, and certainty of the recommendations were low and none except two specified the underlying methodology for the guideline development [7, 8]. Most guidelines lacked evidence grading systems for their recommendations. The recommendations were often very concise, omitting microfilaremia thresholds or drug dosage [16]. Only one of the reviews was a systematic review, which focused rather on symptoms than treatment of loiasis [17]. There is no meta-analysis available which systematically analyses treatment for loiasis.

### Curative treatment recommendations

The identified treatment recommendations showed both similarities and contrasts, particularly in dosage recommendations for diethylcarbamazine, commonly used as a primary treatment across different regions. Most sources recommend initiation of treatment with diethylcarbamazine at a low dose and gradually increasing it, to manage patients effectively and avoid adverse reactions [18, 19]. Typically, diethylcarbamazine full-dose regimens for adults with detected microfilaremia range from 300–400 mg daily [18, 20] to 8–10mg/kg daily [8, 21, 22] over 12 [23] up to 28 days [14, 20], often with repetition of treatment cycles advised if necessary. The starting dose ranges from 3–6 mg daily [10, 24] to 1 mg/kg daily [9, 25–28]. For hypermicrofilaremic patients, the recommended initial dose can be as low as 0.5 mg/kg [22].

In occult loiasis, maintenance doses of diethylcarbamazine range from 400 mg daily [10, 19, 24, 29] to 8–10 mg/kg daily [8, 22] for the same duration as microfilaremic infection. Not all recommendations mention incremental increase of dosing for occult cases, but those who do, recommend initial doses of 50 mg [8, 10, 19, 24, 29] to 6 mg/kg daily [26]. While most sources recommend repetition of the whole treatment cycle in case of persistent infection, another suggestion is treatment for 10 days per month for three to six months, if needed [19]. However, the difficulties in procurement of diethylcarbamazine are also acknowledged [13].

### Alternative treatment recommendations: Ivermectin and albendazole

Ivermectin is another frequently recommended treatment, particularly for patients with a microfilarial load below specific thresholds, highlighting its effectiveness in quickly reducing microfilarial counts [10, 12]. Ivermectin dosing is recommended as alternative treatment when diethylcarbamazine cannot be used, with guidelines recommending a dose of 150 µg/kg [10, 14, 21, 24, 29] to 200 µg/kg [6, 13, 30], most-often as a single-dose [1, 7, 10, 13, 14, 20, 21, 24, 26] but up to a period of ten days in daily doses [6]. Repetitive administrations of ivermectin are considered in some guidelines in monthly [10, 14, 24, 29], quarterly [10, 14, 24, 29] or biannual [13] intervals. Differences arise in frequency and context, such as the Gabonese Health Ministry's recommendation of 200 μg/kg daily for ten days in an endemic setting [6].

Albendazole dosing is commonly prescribed from 400 mg daily [8–12, 14, 15, 18, 20, 21, 24–26, 28, 29] to 800 mg daily [1, 2, 6, 7, 31] for 10 days [6] up to 28 days [1, 7, 15, 31], especially for higher microfilarial loads or when diethylcarbamazine treatment is not feasible [10, 12, 29]. Dosing can be as low as 200 mg three times weekly [25] for low microfilaremia. 800 mg dosing is recommended in case of failure to clear infection by diethylcarbamazine regimens [15]. Rarely, mebendazole is given as an albendazole alternative [17, 26, 31].

### Microfilarial thresholds

Microfilarial thresholds show notable similarities across guidelines, with 8000 microfilariae per millilitre (mf/ml) commonly acknowledged as a critical point for altering treatment strategies. This threshold often prompts more cautious interventions, such as the use of albendazole or apheresis instead of diethylcarbamazine. The threshold recommended for diethylcarbamazine treatment is typically below 1000 mf/ml [8, 15, 18, 20, 25, 26, 28], with some guidelines allowing up to 2000 mf/ml [1, 7, 10, 14, 24, 29]. However, a considerable number of publications set the safe threshold for diethylcarbamazine at 8000 mf/ml [2, 11, 12, 21, 30, 32], with a few sources recommending diethylcarbamazine usage at microfilaremia up to 30,000 mf/ml [22, 33, 34].

For ivermectin therapy, there is more consensus on maintaining the microfilarial threshold below 8000 mf/ml [1, 7, 12, 14, 17, 18, 20, 24–26, 28, 29], although some guidelines still suggest it is safe at levels up to 30,000 mf/ml [10, 19, 29, 33] or only mention elevated risk for Mazzotti reaction above 50,000 mf/ml [13]. The least consensus is seen regarding the microfilarial threshold for albendazole treatment. While there is mostly no upper limit of microfilaremia mentioned, the indication for use of albendazole is varies from > 1000 mf/ml [8, 15] to > 30,000 mf/ml [10, 14, 29], with most recommendations suggesting a threshold of > 8000 mf/ml [6, 11, 12, 17, 18, 20, 21, 25, 26].

Similarly, apheresis is proposed as adjunct treatment strategy for high microfilarial loads of > 8000 mf/ml [11, 12, 25, 26, 30, 32], and at times already at > 2500 mf/ml [9, 35] or for hypermicrofilaremia at > 30,000 mf/ml [1, 7, 22]. Higher thresholds typically signal the necessity for multiple strategies, including inpatient management, to mitigate the risk of severe complications like encephalopathy by close observation of the patient [14, 24, 26, 36].

### Adjunct therapies

Adjunct therapies exhibit notable similarities, with antihistamines and corticosteroids being the most frequently recommended to manage adverse drug effects and improve patient tolerance to treatment regimens with diethylcarbamazine and ivermectin [7, 9, 12]. However, specific recommendations can vary in dosage [6, 19, 21]. While 16 sources made no statement on adjunct therapy and 16 sources suggest possible use of antihistamines and/or corticosteroids, Médecins Sans Frontières Practical Guidelines suggest using paracetamol in case of pain during the initial phase of hypermicrofilaremic treatment and one review mentions reslizumab (anti-interleukin 5) to questionably reduce diethylcarbamazine-induced AEs [14, 21]. While six guidelines propose possible surgical removal for eye migration and 23 make no statement on this matter, Médecins Sans Frontières Practical Guidelines considers this gratuitous, emphasizing non-surgical management approaches. [14, 23],

#### Prophylaxis and mass drug administration

Thirteen of the recommendations suggest the possibility of chemoprophylaxis with diethylcarbamazine in selected high risk travellers, mostly at 300 mg weekly [7, 9, 10, 18, 22–24, 27, 30, 35] to 400 mg on three consecutive days monthly [10, 24], to prevent loiasis, demonstrating a consistent strategy across regions [12, 22]. While four sources suggest alternative dosing, 20 sources do not discuss prophylaxis [19]. Mass drug administration for controlling loiasis in endemic areas is mentioned in the Gabonese guideline, using albendazole and ivermectin to reduce community transmission [6].

## Limitations of the review

The scarcity of guidelines from endemic countries may be attributable to publication bias, whereby health authorities disseminate treatment guidelines through alternative means, such as professional bodies or direct communication, rather than through online publication. This issue could be resolved through direct communication with the relevant authorities. Moreover, the reviewers’ language restriction may have resulted in the omission of sources in Spanish, Portuguese, Arabic, or other languages from endemic countries. This review employed a structured methodology to identify sources that summarise loiasis therapy. A more focused, ideally systematic, review with minimal language restrictions would be beneficial in reliably determining treatment strategies for individual parts of the therapy algorithm, such as through separate reviews of drugs, microfilaremia thresholds, or specific populations.

## Conclusions

The review of clinical management recommendations for loiasis revealed significant variability and inconsistencies across available guidelines, underscoring the challenges faced in achieving effective treatment amidst risks of severe adverse events. Harmonization of guidelines is crucial for improving patient outcomes and establishing safe, evidence-based treatment protocols tailored to diverse healthcare settings.

This review is drawing from a diverse array of sources, including clinical guidelines, reviews, and textbooks. The majority of antiparasitic regimens, including diethylcarbamazine, ivermectin, and albendazole, are recommended as the primary treatment option. Mebendazole is occasionally suggested as an alternative. The higher the microfilarial load, the more likely diethylcarbamazine is to be deferred in favour of ivermectin, then albendazole, and then apheresis. Prophylactic strategies and adjunct therapies, such as corticosteroids and antihistamines, are also frequently proposed, underscoring the necessity of addressing treatment-associated adverse events, particularly in patients with elevated microfilarial loads. It is notable that recommendations vary significantly in detail, dosage, and the microfilarial density categories used to guide treatment selection. This variation encompasses national guidelines from Gabon, the UK and Germany, as well as institutions like the U.S. Centers for Disease Control and Prevention and Médecins Sans Frontières. While there is some consensus globally concerning drugs of choice and need of consideration of microfilarial load, considerable variation in treatment approaches persists. It is notable that the methodological approaches used to formulate these recommendations are often unspecified, which limits the current guidelines' applicability and adaptability. This highlights the need for developing evidence-based guidelines using rigorous methodologies to ensure unbiased recommendations.

### Interpretation of differences

The discrepancies in treatment recommendations for *L. loa* infection might be attributed to a number of factors, including diverse clinical experiences across regions, access to local resources, and differing perspectives from endemic versus non-endemic areas. It is notable that there is considerable variation in the recommended dosage of diethylcarbamazine across different guidelines. This highlights the need for the establishment of universal standards based on robust clinical trials. The methodologies employed for the management of patients with disparate microfilarial loads are inconsistent. Some guidelines recommend adjustments in treatment regimens based on specific microfilarial thresholds, while others lack such criteria. There is considerable variation in the exact thresholds, suggesting uncertainty of when risks are to be expected. Furthermore, the use of adjunct therapies such as antihistamines and corticosteroids is inconsistently addressed. There is also a lack of consensus regarding the prophylactic therapy and management of associated symptoms, such as eye worm migration. Some guidelines advocate for cautious, incremental increases in drug dosage, whereas others provide no such strategies, resulting in inconsistent practices. When discussing diethylcarbamazine, it is crucial to note that diethylcarbamazine is often recommended for use under inpatient conditions in non-endemic settings [7]. However, this may not be feasible in areas with limited healthcare infrastructure, necessitating alternative strategies to ensure safe and effective treatment. The same applies to the availability of apheresis [37]. Finally, as underlying studies for the respective treatment recommendations were mostly not provided in the guidelines, no formal comparison was feasible whether the interpretation of study results was the cause for the differing recommendations.

### Developing robust and adaptive guidelines

In light of the considerable variation in treatment recommendations for *L. loa* infection, there is a pressing need for the development of uniform, evidence-based guidelines that can adapt to diverse health contexts. It is imperative that these guidelines are developed with clear methodologies, including a robust assessment of the risk of bias in the underlying studies and explicit evidence grading. It is essential to define appropriate endpoints and criteria in order to determine the most appropriate course of treatment. This should take into account factors such as microfilaremia, as well as the distinction between endemic and non-endemic areas or high and low resource settings.

There is currently a lack of an agreed-upon definition of cure of loiasis, which complicates the assessment of treatment outcome. As a prerequisite for harmonized treatment guidelines, cure of loiasis as an endpoint of clinical trials should be better defined. Some potential definitions include clinical cure, characterized by the disappearance of symptoms and signs, and parasitological cure, defined as the absence of microfilaremia in the blood. Other indirect indicators, such as the resolution of eosinophilia, decreasing antifilarial antibody titres and the sustained absence of symptoms, may also be used. However, these definitions have significant limitations. Clinical symptoms can be non-specific or absent, and eosinophilia is not observed in all patients. Parasitological cure can be misleading, as macrofilariae might persist despite cleared microfilariae and as accurate detection of microfilariae is not readily available in all endemic settings. Additionally, achieving these endpoints may require multiple treatment courses, complicating the assessment timeline [12, 38–40]. Establishing a clear and clinically relevant definition for cure would aid in standardizing outcomes in research as well as in clinical practice, thereby accelerating the development of more effective treatments and facilitating concise treatment recommendation.

It is imperative that representatives from both endemic and non-endemic areas participate in a global collaboration to harmonise the guidelines. The consideration of resources available in different regions should inform the development of appropriate and locally feasible approaches. The capacity to update guidelines in a dynamic manner through technical solutions such as MAGICapp can facilitate the creation of globally applicable recommendations [41]. It is also imperative that emerging treatments are integrated into these guidelines to ensure their continued relevance.

Although this review is not primarily focused on mass drug administration (MDA) strategies, some of our sources did address this topic. Established MDA campaigns exist for lymphatic filariasis and onchocerciasis, but similar programs for loiasis are not yet in place. Integrating loiasis MDA into broader health initiatives could significantly enhance the overall effectiveness of these programs.

It is also essential to proactively manage policies addressing potential drug unavailability, in the context of loiasis especially regarding diethylcarbamazine. Furthermore, ensuring that healthcare providers are adequately educated and trained in these treatment pathways will further improve patient outcomes. Guidelines should support practitioners in ensuring patient safety and assist in managing severe AEs.

The findings underscore the need to draw up harmonised international guidelines for the effective management of loiasis. By standardising treatment pathways and addressing the complex therapeutic needs of patients, particularly in endemic regions, the risk of severe AEs and fatalities associated with current treatments can be mitigated. Future endeavours should prioritise the development and implementation of evidence-based recommendations that are universally adaptable, thereby ensuring safer and more effective management of this challenging condition.

## Data Availability

Data sharing is not applicable to this article as no datasets were generated or analysed during the current study.

## Abbreviations

AEs: Adverse events
MDA: Mass drug administration
mf/ml: Microfilariae per millilitre
